# Sex differences in the progression of glucose metabolism dysfunction in Alzheimer’s disease

**DOI:** 10.1038/s12276-023-00993-3

**Published:** 2023-05-01

**Authors:** Jong-Chan Park, Hanbyeol Lim, Min Soo Byun, Dahyun Yi, Gihwan Byeon, Gijung Jung, Yu Kyeong Kim, Dong Young Lee, Sun-Ho Han, Inhee Mook-Jung

**Affiliations:** 1 Department of Biophysics, Sungkyunkwan University, Suwon, 16419 Republic of Korea; 2 Institute of Quantum Biophysics, Sungkyunkwan University, Suwon, 16419 Republic of Korea; 3 Department of Medicine, Seoul National University College of Medicine, Seoul, 03080 Republic of Korea; 4 Department of Neuropsychiatry, Seoul National University Hospital, Seoul, 03080 Republic of Korea; 5 Department of Psychiatry, College of Medicine, Seoul National University, Seoul, 03080 Republic of Korea; 6 Department of Nuclear Medicine, SMG-SNU Boramae Medical Center, Seoul, 07061 Republic of Korea; 7 Institute of Human Behavioral Medicine, Medical Research Center, Seoul National University, Seoul, 03080 Republic of Korea; 8 Department of Biochemistry and Biomedical Sciences, College of Medicine, Seoul National University, Seoul, 03080 Republic of Korea; 9 Korea Dementia Research Center, College of Medicine, Seoul National University, Seoul, 03080 Republic of Korea; 10 Ilchun Genomic Medicine Institute (GMI), College of Medicine, Seoul National University, Seoul, 03080 Republic of Korea; 11 Convergence Dementia Research Center, College of Medicine, Seoul National University, Seoul, 03080 Republic of Korea

**Keywords:** Alzheimer's disease, Neurodegeneration

## Abstract

Alzheimer’s disease (AD) is a common neurodegenerative disease characterized by amyloid plaques and impaired brain metabolism. Because women have a higher prevalence of AD than men, sex differences are of great interest. Using cross-sectional and longitudinal data, we showed sex-dependent metabolic dysregulations in the brains of AD patients. Cohort 1 (South Korean, *n* = 181) underwent Pittsburgh compound B-PET, fluorodeoxyglucose-PET, magnetic resonance imaging, and blood biomarker (plasma tau and beta-amyloid 42 and 40) measurements at baseline and two-year follow-ups. Transcriptome analysis of data from Cohorts 2 and 3 (European, *n* = 78; Singaporean, *n* = 18) revealed sex differences in AD-related alterations in brain metabolism. In women (but not in men), all imaging indicators displayed consistent correlation curves with AD progression. At the two-year follow-up, clear brain metabolic impairment was revealed only in women, and the plasma beta-amyloid 42/40 ratio was a possible biomarker for brain metabolism in women. Furthermore, our transcriptome analysis revealed sex differences in transcriptomes and metabolism in the brains of AD patients as well as a molecular network of 25 female-specific glucose metabolic genes (FGGs). We discovered four key-attractor FGG genes (ALDOA, ENO2, PRKACB, and PPP2R5D) that were associated with amyloid/tau-related genes (APP, MAPT, BACE1, and BACE2). Furthermore, these genes successfully distinguished amyloid positivity in women. Understanding sex differences in the pathogenesis of AD and considering these differences will improve development of effective diagnostics and therapeutic treatments for AD.

## Alzheimer’s disease: Metabolic and genetic factors in females

Sex-dependent differences in altered metabolism and gene activity found in patients with Alzheimer’s disease might help reveal why the disease is more prevalent in females than males. Researchers in South Korea led by Inhee Mook-Jung and Sun-Ho Han at Seoul National University found clearly impaired glucose metabolism only in females during a two-year monitoring of 181 South Koreans involving brain scans and blood tests. A separate study of gene activity in 78 European and 18 Singaporean patients identified 25 female-specific genes involved in glucose metabolism. Four of these genes have a role in the events leading to abnormal accumulations of amyloid and tau proteins found in brain cells in Alzheimer’s patients. The results could enhance diagnosis of Alzheimer’s disease and help guide the choice of the most appropriate interventions for different patient groups.

## Introduction

Alzheimer’s disease (AD) is the most prevalent neurological disease and one of the greatest threats to the aging population. Women are more susceptible to AD than men, accounting for almost two-thirds of all cases of AD^1^. Although the average age at onset does not differ between sexes, women are susceptible to more severe clinical and pathological processes, including denser neurofibrillary tangles, greater changes in brain weight, and more severe cognitive dysfunction, than men^2–4^. Moreover, women show more severe clinical dementia than men, as observed on amyloid positron emission tomography (PET) imaging and postmortem brain studies^5,6^.

Changes in brain metabolism can characterize central nervous system diseases^7^; specifically, diverse evidence suggests a close relationship between metabolic dysfunction and Alzheimer’s disease (AD) pathogenesis^8,9^. Patients with AD suffer from both peripheral and central metabolic dysfunction^10,11^; however, some metabolic drugs alleviate AD symptoms^12,13^. In addition, many studies have reported sex differences not only in healthy aging processes but also in AD pathological processes, including brain metabolism, modulation, metabolic connectivity, and networking^14^. Female brains are likely to undergo age-dependent changes and demonstrate hypometabolic phenotypes much earlier than male brains, increasing the risk of AD^15^. Moreover, the significant sex differences in mitochondrial mechanisms and metabolic switches in AD may explain the increased AD risk and distinct disease pathways of women^16^. However, no systemic data or longitudinal results of sex differences in metabolism among patients with AD are available.

This study examined sex differences in metabolic dysregulation in the brains of patients with AD using the results of our previous cross-sectional and longitudinal studies. Moreover, we investigated whether the plasma beta-amyloid (Aβ) 42/40 ratio, a blood biomarker for AD^17,18^, reflects brain metabolic activity and could be used as a potential sex-specific biomarker for metabolic dysfunction in patients with AD. Furthermore, we conducted a transcriptome analysis of the human brain with an independent public cohort and identified sex differences in AD brain transcriptomic and metabolic changes. We believe that the results of this study may inform diagnostic strategies and precision medicine for AD in light of the sex differences.

## Materials and methods

### Participants and ethical approval

Cohort 1 in this study included 181 participants from a longitudinal study. Participant recruitment was based on the protocol from the Korea Brain Aging Study for the Early Diagnosis and Prediction of Alzheimer’s disease (KBASE), and the inclusion and exclusion criteria for the participants have been described in our previous paper^19^. Participants were further divided into PiB-PET-negative (*n* = 127) and PiB-PET-positive groups (*n* = 54) at the baseline timepoint, according to the standardized uptake value ratio (SUVR) value of 1.4 (PiB-PET negative participants had SUVR < 1.4 in all four different regions of interest; PiB-PET positive participants had SUVR > 1.4 in at least one of the four regions of interest; these regions of interest included the frontal, lateral temporal, lateral parietal, and posterior cingulate-precuneus regions)^17,19,20^. Moreover, participants also underwent ^18^F fluorodeoxyglucose-PET (FDG-PET) and magnetic resonance imaging (MRI) using a 3.0-T Biograph mMR (PET-MR) scanner (Siemens, Washington, DC, USA). All participants underwent clinical and neuropsychological assessments and brain imaging twice at the baseline and two-year follow-up timepoints according to the KBASE protocol^19^. Further detailed information on assessments and brain imaging processes is described in our previous report^19,21^. All participants or their legal representatives provided informed consent, and this project was approved by the Institutional Review Board of the Seoul National University Hospital. Cohorts 2 and 3 were public cohorts whose data were extracted for human postmortem brain transcriptome analysis (GEO accession numbers: GSE109887 and GSE150696)^22,23^.

### Measurement of plasma biomarkers

Participants in Cohort 1 visited the hospital at 9 AM, and their blood samples were taken. All fasting blood samples were immediately collected in K2 EDTA tubes (cat: 367855; BD Vacutainer Systems, Plymouth, UK) and centrifuged at 700 × *g* for 5 min at room temperature (RT). Supernatants were collected and centrifuged again to obtain plasma samples. Samples were aliquoted and immediately stored at −80 °C for future use. Total tau plasma levels were measured using Simoa (Total Tau 2.0) kits on the HD-1 Analyzer (cat: 101552; Quanterix, Lexington, MA, USA) at HS Biosystems (Hwaseong, Gyeonggi-do, South Korea) as we previously described^21^. Plasma Aβ levels were quantified using the INNO-BIA plasma Aβ forms kit (cat: 81578; FUJIREBIO, Ghent, Belgium) and Bioplex-200 system (cat: 171000201; Bio-Rad, Hercules, CA, USA) as previously described^17,21^.

### Transcriptome analysis and functional network modeling

For the human brain transcriptome analysis (Cohorts 2 and 3), RNA sequencing data from the public GEO database, containing human brain tissue samples (Cohort 2: 78 samples from the middle temporal gyrus region (32 CN samples and 46 samples with AD); Cohort 3: 18 samples from the prefrontal cortex region (9 CN samples and 9 samples with AD)), were used (GEO accession numbers: GSE109887 and GSE150696)^22,23^. To obtain data on differentially expressed genes (DEGs), the GEO2R analyzer (https://www.ncbi.nlm.nih.gov/geo/geo2r) was used with an FDR-corrected *P* value <0.05. Protein‒protein interaction network and functional enrichment analyses were performed using the STRING database (https://string-db.org) and DEGs from RNA sequencing data. Enriched pathways (KEGG biological processes, molecular functions, and cellular components) were obtained by gene ontology analysis with an FDR-corrected *P* value <0.05. Furthermore, to select metabolic genes or glucose-metabolic genes, the GSEA database was used (GSEA accession number: R-HSA-1430728, R-HSA-70326; https://reactome.org/).

### Statistical analysis

For all analyses, MedCalc version 17.2 software (Ostend, Belgium) or GraphPad Prism version 8 (San Diego, CA, USA) was used. Comparisons between two variables were conducted using an independent-sample t-test. Correlation analyses were performed using Pearson’s correlation analysis or partial correlation analysis with correction for covariates. The sensitivity and specificity of biomarker panels were determined using logistic regression analysis followed by ROC curve analysis. The chi-square test was used to compare categorical variables. In addition, a monotone regression spline analysis was conducted to simulate the gradual progression of AD or brain metabolic dysfunction^24^. This method has been widely used to mimic the longitudinal progression of diseases and reveal biomarker trajectories^24–26^. First, after the variables (plasma biomarkers or brain imaging values) were transformed into z scores (to display and compare them simultaneously within a graph), all curves were generated by the smoothing spline method with four knots using GraphPad Prism 8. At this step, the brain imaging biomarkers also served as a proxy for AD progression (PiB-PET) or brain metabolic dysfunction progression (FDG-PET). Comparison of correlation coefficients (r values) from independent samples from men and women (correcting for the number of patients) was performed according to the Eid, Gollwitzer & Schmidt method as previously reported^27^. Comparisons of spline curve slopes were conducted by GraphPad Prism 8 using spline values from each spline curve, and *p* values for two different slopes were obtained from Deming regression analysis. A comparison of slope analysis was performed using Two Slopes Calculator software (https://www.danielsoper.com/statcalc). Randomized sample selection for women was also performed to balance the numbers of women and men using the randomization function in Microsoft Excel.

### Ethics approval and consent to participate

All participants or their legal representatives provided informed consent, and this project was approved by the Institutional Review Board of the Seoul National University Hospital (E-2009-120-1159).

### Availability of data and materials

All data used in this study are available in the main text or supplementary materials. Human brain transcriptome data (Cohort 2) consisted of RNA sequencing data from the public GEO database, comprising brain tissue samples (78 from the middle temporal gyrus region; 32 CN samples and 46 AD samples), were used (GEO accession number: GSE109887). Additional human brain transcriptome data (Cohort 3) consisted of RNA sequencing data from the public GEO database, comprising brain tissue samples (18 from the prefrontal cortex region; 9 CN samples and 9 samples with AD), were used (GEO accession number: GSE150696).

## Results

### Overall study procedures and demographic characteristics of participants

**Fig. 1:**
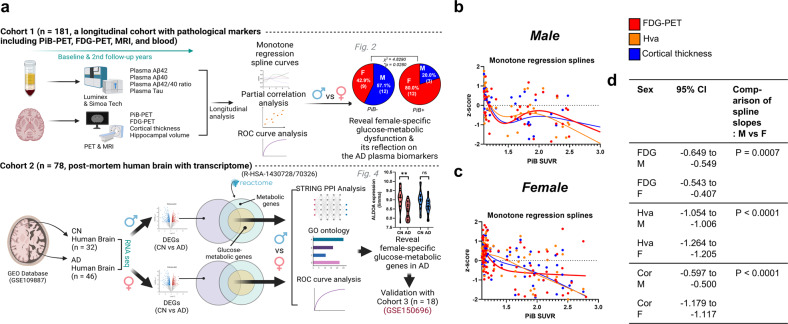
Sex differences in imaging and plasma biomarkers through monotone regression splines. **a** Overall experimental procedure. Two independent cohorts (Cohort 1, South Korean, *n* = 181; Cohort 2, European, *n* = 78) were used for this study. Cohort 1 underwent Pittsburgh compound B (PiB)-positron emission tomography (PET), fluorodeoxyglucose (FDG)-PET, magnetic resonance imaging, and blood biomarker (plasma tau and beta-amyloid 42 and 40) quantification at both baseline and at a two-year follow-up. Cohort 2 was included in a transcriptome analysis (GEO Database, GSE 109887) using human postmortem brain samples. Both analyses using Cohorts 1 and 2 had the same objective of revealing female-specific glucose metabolism changes in AD. Another publicly available dataset from the GEO database (Cohort 3, GSE150696) was also used to validate the results from Cohort 2. **b**, **c** Monotone spline models for brain imaging biomarkers in both men and women. PiB-PET SUVR was used as a proxy for the progression time of AD. To effectively show comparisons between different imaging biomarkers, their levels were transformed to z scores. **d** Comparison of spline slope analysis between women and men. AD Alzheimer’s disease, PiB-PET Pittsburgh compound B-positron emission tomography, SUVR standardized uptake value ratio, Hva hippocampal volume, FDG-PET fluorodeoxyglucose PET, 95% CI 95% confidence interval.

The flow chart of the experimental procedures is presented in Fig. 1a. Three independent cohorts (Cohort 1, South Korean cohort for longitudinal analysis of brain imaging results and plasma biomarkers [*n* = 181]; Cohort 2, European cohort for brain transcriptome analysis [*n* = 78]; Cohort 3, Singaporean cohort for brain transcriptome analysis [*n* = 18]) were included in this study. Cohort 1 included 181 participants (men: 46 Pittsburgh compound B [PiB]-PET-negative and 23 PiB-PET-positive; women; 81 PiB-PET-negative and 31 PiB-PET-positive) at both baseline and two-year follow-up timepoints. There were no significant sex differences in patient characteristics, except for PiB-SUVR values within the PiB-PET-negative group, implying that women may have had higher cerebral amyloid depositions than men despite being included in the PiB-PET-negative group (standardized uptake value ratio; SUVR < 1.4). Cohort 2 included 78 participants (men: 16 cognitively normal [CN] and 22 with AD; women: 16 CN and 24 with AD). Cohort 3 included 18 participants (men: 4 CN and 4 with AD; women: 5 CN and 5 with AD) (Table 1).

**Table 1:** Demographic characteristics of participants (*n* = 181 for Cohort 1; *n* = 78 for Cohort 2; *n* = 18 for Cohort 3).

Cohort 1	Characteristic	PiB-PET negative (*n* = 127)			PiB-PET positive (*n* = 54)		
		M (*n* = 46)	F (*n* = 81)	*P* value	M (*n* = 23)	F (*n* = 31)	*P* value
Baseline	Global PiB SUVR, mean ± SEM	1.09 ± 0.009	1.11 ± 0.009	0.0417a	1.78 ± 0.060	1.93 ± 0.067	0.1144a
Two-year follow-up		1.08 ± 0.009	1.11 ± 0.085	0.0243a	1.98 ± 0.088	2.06 ± 0.069	0.4788a
Baseline	FDG SUVR (4 ROIs), mean ± SEM	1.51 ± 0.021	1.49 ± 0.014	0.3841a	1.38 ± 0.027	1.39 ± 0.025	0.8089a
Two-year follow-up		1.44 ± 0.020	1.46 ± 0.017	0.6099a	1.33 ± 0.028	1.29 ± 0.034	0.4346a
Baseline	CN/MCI/AD, (n)	41/5/0	69/7/5	0.2180b	11/6/5	9/11/11	0.2918b
Two-year follow-up		42/4/0	67/7/7	0.1208b	11/4/7	9/9/13	0.2914b
Baseline	FDG-positive, n/N (%)	21/46 (46%)	36/81 (44%)	0.8958b	18/23 (78%)	21/31 (68%)	0.3979b
Two-year follow-up		31/46 (67%)	46/81 (57%)	0.2417b	18/23 (78%)	24/31 (77%)	0.9419b
Baseline	Age (years), mean ± SEM	69.78 ± 1.029	68.12 ± 0.889	0.2423a	74.09 ± 1.372	71.45 ± 1.095	0.1349a
Two-year follow-up		72.07 ± 1.033	70.38 ± 0.878	0.2320a	76.35 ± 1.343	73.58 ± 1.108	0.1158a
Baseline	CDR 0/0.5/1/2 (n)	41/5/0/0	69/8/4/0	0.3084b	12/9/2/0	9/16/6/0	0.2580b
Two-year follow-up		41/5/0/0	67/7/7/0	0.1182b	12/4/6/1	9/7/11/4	0.3330b
Baseline	MMSE z score, mean ± SEM	0.18 ± 0.131	−0.01 ± 0.114	0.2789a	−1.50 ± 0.439	−1.71 ± 0.287	0.6793a
Two-year follow-up		0.20 ± 0.133	0.12 ± 0.125	0.7131a	−1.48 ± 0.512	−2.18 ± 0.387	0.2705a
Baseline	ApoE4-positive, n/N (%)	9/46 (20%)	8/81 (10%)	0.1247b	9/23 (39%)	19/31 (61%)	0.1104b
Two-year follow-up		9/46 (20%)	8/81 (10%)	0.1247b	9/23 (39%)	19/31 (61%)	0.1104b

Cohort 2 (GSE109887)	Group	Source name	Sex	Mean age, years	Tissue	Disease
*n* = 24	AD_F	Middle temporal gyrus, AD	Female	85.5	Brain, middle temporal gyrus	AD
*n* = 22	AD_M	Middle temporal gyrus, AD	Male	84.6	Brain, middle temporal gyrus	AD
*n* = 16	CN_F	Middle temporal gyrus, CN	Female	84.2	Brain, middle temporal gyrus	CN
*n* = 16	CN_M	Middle temporal gyrus, CN	Male	85.0	Brain, middle temporal gyrus	CN

Cohort 3 (GSE150696)	Group	Source name	Sex	Mean age, years	Tissue	Disease
*n* = 5	AD_F	Prefrontal cortex, AD	Female	83.2	Brain, prefrontal cortex	AD
*n* = 4	AD_M	Prefrontal cortex, AD	Male	88.75	Brain, prefrontal cortex	AD
*n* = 5	CN_F	Prefrontal cortex, CN	Female	86.8	Brain, prefrontal cortex	CN
*n* = 4	CN_M	Prefrontal cortex, CN	Male	82.0	Brain, prefrontal cortex	CN

The *P* values were obtained by (a) independent-sample t tests or (b) chi-square tests.

*PiB* Pittsburgh compound B, *FDG* fluorodeoxyglucose, *CDR* clinical dementia rating, *MMSE* Mini-Mental State Examination, *MMSE z score* Mini-Mental State Examination score with adjustment for covariates (age, sex, and educational level), *ApoE* Apolipoprotein E, *SEM* standard error of mean, *ROI* region of interest, *SUVR* standardized uptake value ratio.

### Monotone regression splines reveal potential sex differences in AD

To understand sex differences in AD, we investigated whether imaging or plasma biomarker data displayed sex differences in trends of disease progression. Accordingly, we performed monotone penalized regression spline analyses^24^ to identify the relationship between each biomarker and imaging variable. In women (but not in men), all imaging variables reflecting brain hypometabolism (on FDG-PET scans) or neurodegeneration (i.e., hippocampal volume changes and decreases in cortical thickness) had stable correlations with AD progression (PiB-PET SUVR; a proxy for progression time) (Fig. 1b–d). In addition, each graph was divided into three zones according to the degree of cerebral amyloid deposition (zone 1, PiB-PET SUVR < 1.4; zone 2, 1.4 ≤ PiB-PET SUVR < 2.0; and zone 3, PiB-PET SUVR ≥ 2.0). Interestingly, we also observed different patterns between women and men in only zone 2 (SUVR = 1.4–2.0); there were noticeable opposite trends between men and women in FDG-PET and hippocampal volume changes (Supplementary Fig. 1). This phenomenon motivated us to study sex differences in the progression of AD in greater detail. Subsequently, we investigated which plasma biomarkers indicated sex differences in AD. Among the monotone regression splines with representative plasma biomarkers for AD (Aβ42, Aβ40, Aβ42/40 ratio, and total tau), the plasma Aβ42/40 ratio had a stable relationship with the progression of imaging biomarkers in women (except for slight fluctuation in cortical thickness at the initial stage), whereas there were no noticeable stable biomarkers in men (Supplementary Fig. 2). Even though the plasma Aβ42/40 ratio in men showed a clear correlation with FDG-PET SUVR values (Supplementary Fig. 2a, first graph), we believe that it would not be a good biomarker in men as (i) FDG-PET SUVR values did not show a smooth correlation with PiB-PET SUVR values (Fig. 1b) and (ii) the plasma Aβ42/40 ratio exhibited a quadratic relationship with PiB-PET SUVR values (Supplementary Fig. 2a, lower left graph) in men, in contrast to that in women (Supplementary Fig. 2b). There may be a large sex difference in brain metabolism represented by FDG-PET imaging when controlling for PiB-PET status during AD progression. Accordingly, we concluded that evaluating brain metabolism as reflected in the plasma Aβ42/40 ratio may identify sex differences in AD.

### Sex differences in the progression of brain hypometabolism in AD

**Fig. 2:**
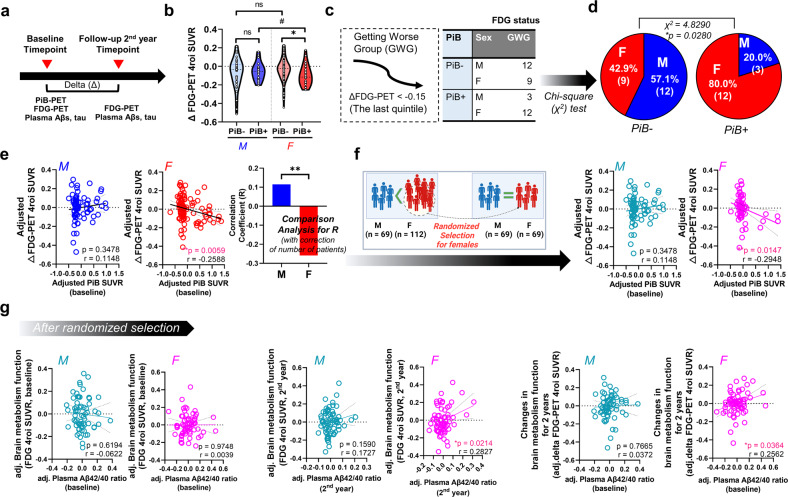
Sex differences in the progression of brain hypometabolism in patients with AD and the relationship with the plasma beta-amyloid 42/40 ratio. **a** Longitudinal study timeline. **b** Comparison of brain metabolism function at baseline and at a two-year follow-up between sexes. #*P* < 0.1 and *P < 0.05 as assessed using the two-sided independent-sample *t* test. **c** Patients in the last quintile (delta FDG-PET < −0.15 at the two-year follow-up) were placed in the worsening group. **d** Results of a chi-square test to compare the PiB-PET-negative (PiB–) and PiB-PET-positive (PiB+) subgroups of the worsening group (Getting worse group). There were more women in the PiB+ subgroup than in the PiB– subgroup. *P < 0.05 as assessed by the chi-square test. Delta indicates the difference between the first (baseline) and second (two-year follow-up) measurement values. **e** Sex differences in correlation patterns of brain metabolic status with cerebral amyloid deposition. **P < 0.01 as assessed using partial correlation analysis with age as a covariate. Correlation analysis corrected for the number of patients was also performed (**P < 0.01). **f** Sex differences in correlation patterns of brain metabolic status with cerebral amyloid deposition after performing randomized selection for women (*P < 0.05). **g** Sex differences in correlation patterns. Partial correlation analyses between the plasma beta-amyloid 42/40 ratio and FDG-PET SUVR (4 ROIs) after randomized selection of women were conducted. PiB-PET SUVR values and age were included as covariates. Delta indicates the difference between the first and second measurement values (*P < 0.05). See Supplementary Fig. 3 for all comparisons. Abbreviations: PiB-*P*ET, Pittsburgh compound B-positron emission tomography; SUVR, standardized uptake value ratio; adj, adjusted.

Figure 2a presents the timeline of our longitudinal study. Delta values indicate differences between the first (baseline) and second (2-year follow-up) values (e.g., delta FDG-PET values and changes in brain metabolism function for 2 years). When we compared delta FDG-PET values between PiB− and PiB+ patients among both sexes, patients with PiB+ had significantly lower delta FDG-PET values than patients with PiB− among women but not men (Fig. 2b). Moreover, the PiB+ female group had significantly lower delta FDG-PET values than the PiB+ male group (Fig. 2b). Subsequently, we categorized patients with severe symptoms (i.e., patients in the last and lowest quintile of delta FDG-PET values) into the worsening group (12 men with PiB− and 9 women with PiB−; 3 men with PiB+ and 12 women with PiB+). Moreover, we calculated the sex ratios of both the PiB− and PiB+ groups. Interestingly, the PiB+ subgroup of the worsening group had significantly more women than men (Fig. 2c, d). Partial correlation analysis (corrected for the number of patients) further showed that delta FDG-PET SUVR was correlated with PiB-PET SUVR in women but not men (Fig. 2e). This tendency was true even after performing randomized sample selection for women (Fig. 2f), implying the presence of sex differences in brain metabolism progression in patients with AD, although there were seemingly no sex differences in FDG-PET SUVR from our superficial observation of Table 1.

### The plasma Aβ42/40 ratio is an indicator of brain metabolic dysfunction in women

Since our monotone regression splines revealed that the plasma Aβ42/40 ratio had the best fit with FDG-PET SUVR values in women but not in men and considering the curve pattern of the plasma Aβ42/40 ratio regarding PiB-PET SUVR progression, we performed a partial correlation analysis between this biomarker and FDG-PET SUVR values with adjustments for PiB-PET SUVR and age (Fig. 2g and Supplementary Fig. 3). As expected, FDG-PET values had significant positive correlations with the plasma Aβ42/40 ratio in women both cross-sectionally and longitudinally, even after adjusting for covariates (PiB-PET SUVR and age) (Fig. 2g) and performing randomized selection for women. Moreover, partial correlation analysis (Supplementary Fig. 3) (corrected for the number of patients) further showed significant sex differences in the correlation of delta FDG-PET (Supplementary Fig. 3). Nevertheless, there was not a significant correlation between the plasma Aβ42/40 ratio and hippocampal volume or cortical thickness when controlling for covariates (Supplementary Table 1), again suggesting that the plasma Aβ42/40 ratio is a strong candidate indicator for brain metabolic dysfunction in women but not in men.

### Other plasma biomarkers showing partial correlations with brain metabolic dysfunction

We further performed partial correlation analyses to identify possible links between other plasma biomarkers (Aβ42, Aβ40, and total tau levels) and brain imaging markers (Supplementary Tables 2–3). There were several correlations between plasma Aβ42 or Aβ40 levels and FDG regions of interest (ROIs) in women but not in men (Supplementary Table 2). These biomarkers also had weak correlations with other neurodegeneration markers (i.e., hippocampal volume and cortical thickness) in women (Supplementary Table 3). However, unexpectedly, plasma tau levels had partial correlations with some FDG ROIs (integrated 4 ROIs, hippocampus, and inferior temporal gyrus) in men (Supplementary Table 2). This is possibly due to the curve of plasma tau levels in the monotone splines for men (orange line, convex downward shape) related to FDG-PET SUVR (Supplementary Fig. 2a, first graph); this curve was clearly opposite that of plasma tau levels (orange line, convex upward shape) related to PiB-PET SUVR (Supplementary Fig. 2a, lower left graph).

### Use of the plasma Aβ42/40 ratio for diagnostic or predictive models of brain metabolic dysfunction

To evaluate the potential use of the plasma Aβ42/40 ratio in diagnosing or predicting brain metabolic dysfunction, we performed logistic regression analyses comprising receiver-operating characteristic (ROC) curve analyses (Supplementary Fig. 4). For the diagnostic model, we included the plasma Aβ42/40 ratio at the two-year follow-up as a dependent variable and the covariates of age, education levels, and apolipoprotein E genotype. FDG-PET positivity (negative or positive, at the two-year follow-up), the independent variable in this model, was defined according to a previous study^28^. We confirmed that the plasma Aβ42/40 ratio had higher discriminative power in women (area under the curve [AUC] = 0.712, with a sensitivity of 75.0% and a specificity of 62.3%) than in men ([AUC] = 0.610, with a sensitivity of 81.6% and a specificity of 46.7%) when including covariates (Supplementary Fig. 4a). For the predictive model, we included the plasma Aβ42/40 ratio at baseline as the dependent variable and the covariates of age, education levels, and apolipoprotein E genotype. The worsening status of the patient (positive, delta FDG < −0.15; negative, delta FDG > −0.15; Fig. 2) was included as an independent variable. Moreover, we verified that the plasma Aβ42/40 ratio showed higher discriminative power in women ([AUC] = 0.719, with a sensitivity of 85.0% and a specificity of 58.9%) than in men ([AUC] = 0.709, with a sensitivity of 85.7% and a sensitivity of 65.4%) (Supplementary Fig. 4b). Thus, we suggest that the plasma Aβ42/40 ratio is a potential biomarker that can be included in diagnostic or predictive models to determine brain metabolic status.

### Human brain transcriptomic analysis revealed female-specific regulation of glucose-metabolic genes in patients with AD

**Fig. 3:**
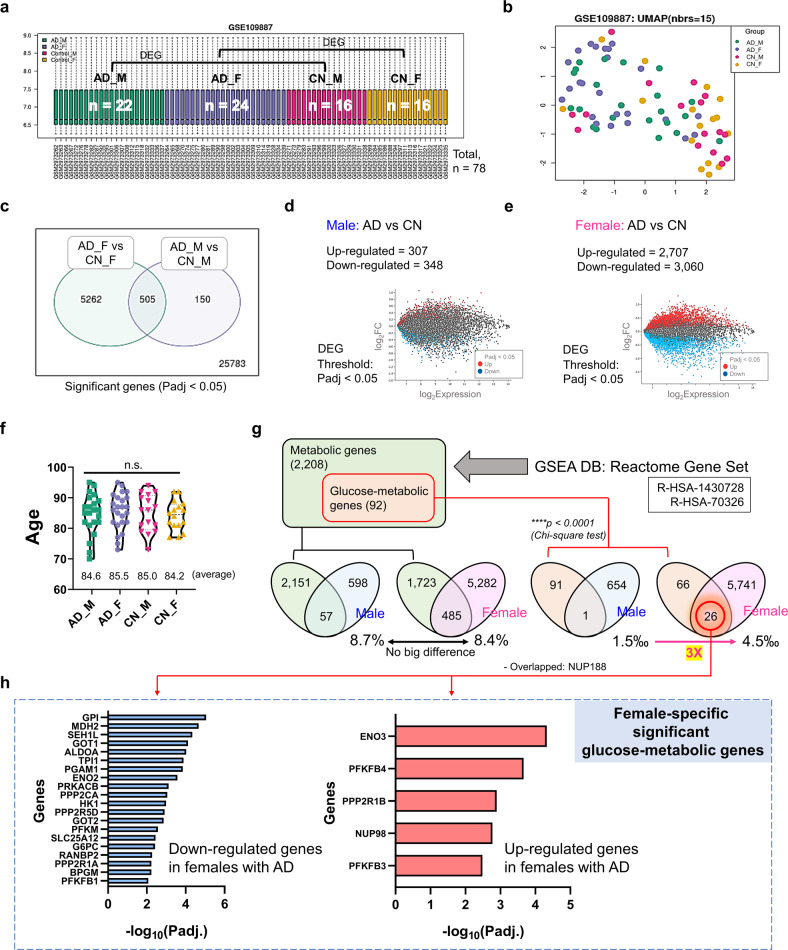
Sex differences in the AD brain (middle temporal gyrus) in transcriptomic and metabolic changes. **a** Gene expression distribution after normalization for RNA sequencing (boxplots show 3rd quartile + 1.5 IQR with upper whiskers and 1st quartile – 1.5 IQR of lower whiskers; Q1 (25th percentile) and Q3 (75th percentile) serve as the box bounds and Q2 (50th percentile) is the center bold line) of human brain (middle temporal gyrus) transcriptome data (16 CN men, 16 CN men, 22 men with AD, and 24 women with AD; *n* = 78) from the GEO2R public database (accession number: GSE109887; platform number: GPL10904). **b** Principal component analysis (PCA) plot showing transcriptomic expression patterns in RNA sequencing data. **c** The number of overlapping DEGs in male and female patients with AD. DEG threshold, FDR-adjusted *P* < 0.05. **d** The number of DEGs in male patients with AD. **e** The number of DEGs in female patients with AD. **f** There were no differences in age among groups. *P* values were obtained using a one-way ANOVA with post hoc tests. **g** The number of DEGs overlapping with metabolic genes or glucose-specific metabolic genes. Metabolic genes were selected by GSEA in the reactome gene set (R-HSA-1430728, R-HSA-70326). **h** Downregulated (20) and upregulated DEGs (6) in women with AD. F female, M male, Padj FDR-adjusted *P* value, DEG differentially expressed gene, GSEA gene-set enrichment analysis, DB database, CN cognitively normal.

**Fig. 4:**
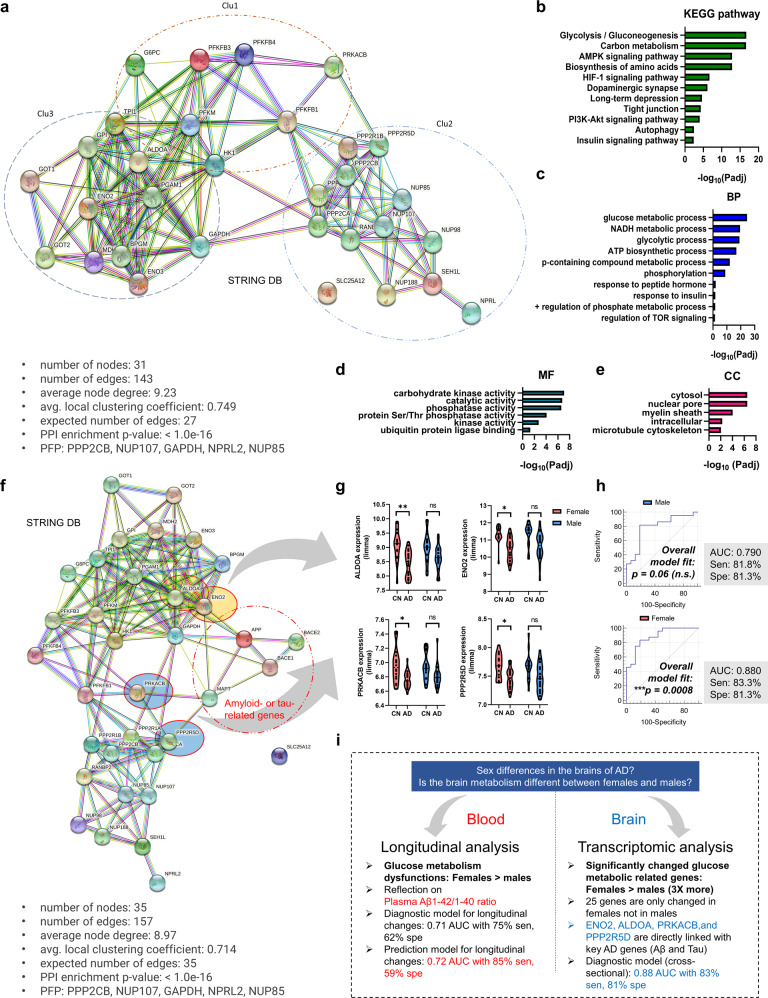
Molecular network of a glucose-metabolic gene set specifically regulated in female AD patients (*n* = 25). **a** A functional enrichment analysis to reveal protein‒protein interaction networks (31 nodes). Five proteins (PPP2CB, NUP107, GAPDH, NPRL2, and NUP85) were used as predicted functional partners (PFP) in the network model. Three clusters were detected by K-means clustering analysis. Colored nodes: query proteins and first shell of interactors; white nodes: second shell of interactors; filled nodes: some three-dimensional structures known or predicted; edges: protein‒protein associations. **b**–**e** Enriched KEGG pathways and gene ontology analysis (BP, CC, MF) using glucose metabolic genes specifically regulated in women. The analytic threshold of enrichment analyses was an FDR-adjusted *P* < 0.05. **f** A functional enrichment analysis to reveal protein‒protein interaction networks with key AD-related biomarker genes (APP, BACE1, BACE2, and MAPT; amyloid- or tau-related genes) (36 nodes). Five proteins (PPP2CB, NUP107, GAPDH, NPRL2, and NUP85) were used as predicted functional partners (PFP) in the network model. Colored nodes: query proteins and first shell of interactors; white nodes: second shell of interactors; filled nodes: some three-dimensional structures known or predicted; edges: protein‒protein associations. **g** mRNA expression of genes directly linked with key AD genes. Two-way ANOVA with Tukey’s post hoc test (cutoff: *P* < 0.01). **h** ROC curve analysis using the ALDOA, ENO2, PRKACB, and PPP2R5D genes. The logistic regression analysis showed significant results for women but not men. **i** Graphical summary of this study. Padj FDR-adjusted *P* values, DEG differentially expressed genes, BP biological process, CC cellular components, MF molecular functions, PFP predicted functional partners.

To investigate which specific molecular factors contribute to brain metabolic dysfunction in the female brain, an additional transcriptome analysis using human brain (middle temporal gyrus) samples was performed using the GEO2R analyzer (https://www.ncbi.nlm.nih.gov/geo/geo2r) and an independent public cohort (Cohort 2 with GEO accession number GSE109887 [*n* = 78]; Figs. 3 and 4). This dataset is very well normalized and readily included in differential expression analyses (Fig. 3a, Supplementary Fig. 5). The principal component analysis plot showed distinct disease-dependent separation between CN individuals and patients with AD (Fig. 3b). Interestingly, the number of differentially expressed genes (DEGs) in female patients with AD was 8.8 times higher than that in male patients with AD (655 male DEGs and 5767 female DEGs) (Fig. 3c–e), although the total number of read genes, numbers of participants (men: 16 CN and 22 with AD; women: 16 CN and 24 with AD; Fig. 3a), and average age (male CN participants, 85.0 years; male patients with AD, 84.6 years; female CN participants, 84.2 years; and female patients with AD, 85.5 years; Fig. 3f) were similar. Subsequently, to determine how many of these DEGs were metabolic-related genes, especially glucose-metabolic genes, these genes were selected using a gene-set enrichment analysis (GSEA) database (accession numbers: R-HSA-1430728 and R-HSA-70326), and the degree of overlap among DEGs was tested (Fig. 3g). Interestingly, the male AD DEGs included only one glucose-metabolic gene (NUP188), whereas the AD female DEGs included 26 glucose-metabolic genes (GOT1, ENO2, GPI, PGAM1, and so on) (Figs. 3g and 4a). The proportion of glucose-metabolic genes per whole DEG for each sex was 3 times higher in women (4.5‰) than in men (1.5‰), despite the difference in the total number of DEGs between men and women (Fig. 3g). The female-specific downregulated (20 genes) and upregulated DEGs (5 genes) in female patients with AD are presented in Fig. 3h. Among the 26 female DEGs, NUP188 was the only gene that showed a significant difference among men with AD. We further validated the results from Cohort 2 using another independent cohort (Cohort 3, publicly available in GEO database; GSE150696) (Supplementary Fig. 6a–c). This dataset was also very well normalized and readily included in differential expression analyses (Supplementary Fig. 6b, left graph), and the principal component analysis plot showed distinct disease-dependent separation between CN individuals and patients with AD (Supplementary Fig. 6b, right graph). Interestingly, the up/downregulated tendency of DEGs in women from Cohort 3 was highly similar to the results in women from Cohort 2 (equality, 84%) (Supplementary Fig. 6d). In addition, we observed that the female-specific glucose metabolic genes from Cohort 2 also significantly differed between CN and AD participants in women but not in men (Supplementary Fig. 6e).

### Molecular network of female-specific glucose-metabolic gene sets and its potential pathways

We found glucose-metabolic genes specifically regulated in women, but it remained unclear which biological pathways were related to metabolic dysregulation in female patients with AD and how these metabolic genes were interconnected. Using the STRING database, an analytic tool for functional protein association networks, we established a molecular network of glucose-metabolic gene sets specifically regulated in women (Fig. 4a). Interestingly, there were three distinct K-mean clusters, and all clusters were well-balanced with appropriate up- and downregulated metabolic genes. The molecular network identified significant relevant KEGG pathways (FDR-adjusted *P* value <0.05) crucial for brain health as well as glycolysis, gluconeogenesis, carbon metabolism, dopaminergic synapse, autophagy, insulin signaling pathway, and so on (Fig. 4b). Moreover, a gene ontology (GO) analysis showed that several key biological processes (BP), cellular components (CC), and molecular functions (MF) were linked to the aforementioned molecular network (Fig. 4c–e). Subsequently, to identify attractor genes directly linked with the expression of amyloid- or tau-related genes (APP, BACE1, BACE2, and MAPT) associated with AD, we again established a molecular network of glucose-metabolic gene sets specifically regulated in women comprising these genes (Fig. 4f). Interestingly, aldolase and neuron-specific enolase were directly associated with the APP gene; moreover, protein kinase cyclic AMP (cAMP)-activated catalytic subunit beta (PRKACB) and protein phosphatase 2 regulatory subunit B delta (PPP2R5D) were associated with the MAPT genes. As expected, these genes were downregulated in women with AD but not in men with AD (Fig. 4g). The ROC curve analysis followed by logistic regressions comprising these four genes showed a significant overall fit only in women (Fig. 4h). The overall results of this study are summarized in Fig. 4i.

## Discussion

Sex differences can be derived from environmental factors, including social, cultural, and exposure factors, although sex differences are based on biological properties^29,30^. To objectively analyze sex differences in AD using our Korean cohort, Cohort 1, we considered the following critical points when comparing sexes. First, to minimize the environmental factors that may have affected participant examination, we collected blood samples from all fasted participants at 9:00 AM and guided them to undergo neuroimaging in the afternoon of the same day to ensure the same conditions. Second, all study participants underwent various neuroimaging examinations, such as PiB-PET, FDG-PET, and MRI, to extensively address neuropathologic changes and direct changes in cerebral amyloid deposition in the brain. Since we did not find any noticeable cross-sectional differences between men and women (Supplementary Table 1), we further investigated longitudinal changes in neuropathological traits between sexes at the two-year follow-up. Monotone spline curve analyses demonstrated different tendencies between sexes, including in plasma biomarker and neuroimaging data (Fig. 1). We speculate that these differences are associated with sex differences in neural or cognitive reserves (i.e., resistance to brain damage or impairment of cognitive function during the progression of dementia)^31^; numerous reports have shown a higher incidence rate of dementia in women than in men and suggested the relevance of neural/cognitive reserves to sex differences^29–32^. Third, when proceeding with the analysis, we considered as many factors that may influenced the results as possible. For example, since there is a clear difference in lifespan between sexes, we adjusted for age in all correlation analyses (Fig. 2c, Supplementary Fig. 4, Supplementary Tables 1–3). Moreover, whenever we compared FDG-PET SUVR values with plasma biomarkers, such as Aβ1-42 and 1-40 levels, the 42/40 ratio, and t-tau, we corrected for PiB-PET SUVR values to remove their influence (Fig. 2, Supplementary Tables 2–4). Finally, we identified distinct sex differences in the progression of brain glucose metabolism dysfunction in patients with AD using these stringent control procedures and numerous longitudinal analytical results indicated that men lack a significant correlation of FDG-PET and PiB-PET variables with plasma biomarker levels, in contrast to the findings in women (Fig. 2e–g, and Supplementary Fig. 3). As shown in Fig. 2e and Supplementary Fig. 4, power analysis indicated that there was considerable power for analyses in women but that those in men were underpowered, indicating that women have much more significant correlations of FDG-PET and PiB-PET variables with FDG-PET variables and the plasma beta-amyloid 42/40 ratio. When a statistical analysis is referred to as ‘underpowered,’ vastly increasing the sample size may detect a significant specific outcome (correlation coefficient (r) or AUC value). However, our goal was not to collect enough male patients to alleviate the ‘underpowered’ nature of these analyses but rather to identify nonsignificant results for men and significant results for women. We believe there are effects in women but not in men because we thoroughly confirmed that the statistical analyses (partial correlation analysis or ROC curve analysis) were performed correctly with appropriate covariates or outlier tests, although the statistical power analysis for men was ‘underpowered’. Next, we validated our hypothesis using a transcriptome analysis with two independent cohorts (Cohorts 2 and 3 from the GEO database), clearly identifying biological pathways relevant to metabolic dysregulation in patients with AD and how these critical genes are associated with each other (Figs. 3 and 4). Although the analysis is independent of that from Cohort 1 and does not indicate replicative validation, we strongly believe that it is a meaningful external validation in that it (i) utilized the transcriptome datasets from the human postmortem brain samples, (ii) indicated novel female-specific glucose-metabolic genes, and (iii) had the same objective (to identify female-specific glucose metabolic changes in AD). Similar approaches using 2–3 independent cohorts but performing different types of analyses are common in other cohort-based studies^33,34^.

In summary, our current study presents novel insight into sex differences among patients with AD. Moreover, to our knowledge, this study is the first to reveal that the plasma Aβ42/40 ratio is a strong candidate indicator for brain metabolic dysfunction in women but not in men (Figs. 1, 2 and Supplementary Fig. 3). Moreover, through including the plasma Aβ42/40 ratio in the ROC curve analysis to diagnose or predict brain hypometabolism (FDG-PET positivity), we showed that well-known plasma biomarkers, such as the Aβ42/40 ratio^17,35,36^, could also provide differential information according to sex(Supplementary Fig. 4), indicating that sex differences in biomarkers should be studied to truly understand sex-dependent neuropathological changes in patients with AD. Moreover, we identified a molecular network for female patients with AD and validated possible factors that trigger sex differences in brain hypometabolism progression (Figs. 3 and 4).

This study has some limitations. Although we identified possible factors influencing sex differences using our longitudinal study, further studies with more patients and longer follow-up periods are needed. In addition, since Cohorts 1 and 2 were completely independent cohorts and drawn from different ethnic groups (Cohort 1, South Korean cohort from Seoul National University Hospital; Cohort 2, German cohort from University Medical Centre Gottingen), further validations of each cohort with participants of similar ethnicity are needed. We cannot rule out the possibilities of interracial differences in environmental backgrounds, dietary habits, and cultures. With further validation, however, we believe that it would be possible to develop precision medicine that can be applied differently to male and female patients with AD because both cohorts showed similar results related to female-specific glucose metabolic changes in AD.

In conclusion, our longitudinal and transcriptome analyses clearly revealed sex differences in human metabolic changes associated with AD and identified 25 novel female-specific glucose metabolic genes. We offer crucial evidence on sex differences in pathological AD mechanisms and suggest that these differences can help to elucidate the pathophysiology of AD and develop appropriate diagnostics and therapeutic treatments for AD.

## Supplementary information

Supplemental_materials
