# Supplementary material for: Sex differences in the progression of glucose metabolism dysfunction in Alzheimer’s disease
Source: Exp Mol Med. 2023 May 1;55(5):1023–32. doi: 10.1038/s12276-023-00993-3 (PMC10238450; doi:10.1038/s12276-023-00993-3)
Supplement: Supplementary file 1 — Supplemental_materials [file 12276_2023_993_MOESM1_ESM.pdf]

Experimental & Molecular Medicine

Supplemental materials

TITLE: Sex differences in the progression of glucose metabolism dysfunction in Alzheimer's disease

Park *et al.*

***Corresponding authors:**

1) Inhee Mook-Jung, Ph.D.

Department of Biochemistry & Biomedical Sciences, SNU Dementia Research Center

Seoul National University College of Medicine

103 Daehak-ro, Jongno-gu, Seoul 03080, Republic of Korea

Tel: +82-2-740-8245; Fax: +82-2-3672-7352; Email: inhee@snu.ac.kr

2) Sun-Ho Han, Ph.D.

Department of Biochemistry & Biomedical Sciences, Neuroscience Research Institute

Seoul National University College of Medicine

103 Daehak-ro, Jongno-gu, Seoul 03080, Republic of Korea

Tel: +82-2-3668-7640; Fax: +82-2-3672-7352; Email: sunho@snu.ac.kr

Supplementary Tables

Supplementary Table 1. Neurodegeneration markers and plasma beta-amyloid 42/40 ratio

MRI	Gender	Plasma beta-amyloid 42/40 ratio<br>Neurodegeneration (baseline) vs beta-amyloid 42/40 (baseline)	Neurodegeneration (2nd year) vs beta-amyloid 42/40 (2nd year)	Δ Neurodegeneration vs beta-amyloid 42/40 (baseline)
**Hippocampal volume**	**Male**	p = 0.9827<br>r = -0.0028	p = 0.8296<br>r = -0.0270	p = 0.5298<br>r = -0.0820
	**Female**	p = 0.7970<br>r = 0.0265	p = 0.4530<br>r = 0.0737	p = 0.9291<br>r = -0.0092
**Cortical thickness**	**Male**	p = 0.4100<br>r = 0.1065	p = 0.1885<br>r = 0.1639	p = 0.3681<br>r = 0.1163
	**Female**	***#p = 0.0604***<br>***r = 0.1866***	p = 0.7686<br>r = 0.0289	p = 0.4188<br>r = -0.0809

- The statistical p-values were obtained by partial correlation analysis (covariates: age, PiB-PET SUVR values; #p < 0.1, *p < 0.05, and **p < 0.01). Statistically significant p-values were shown in bold & italic.
- Abbreviations: MRI, magnetic resonance imaging; delta (Δ), difference between the second and first measurement values

Supplementary Table 2. Different correlation patterns of brain metabolism with other representative plasma AD biomarkers between males and females

		**Plasma beta-amyloid 1-42**			**Plasma beta-amyloid 1-40**			**Plasma tau**		
**Brain ROIs**	**Gender**	**FDG (baseline) vs beta-amyloid 1-42 (baseline)**	**FDG (2nd year) vs beta-amyloid 1-42 (2nd year)**	**Δ FDG (delta) vs beta-amyloid 1-42 (baseline)**	**FDG (baseline) vs beta-amyloid 1-40 (baseline)**	**FDG (2nd year) vs beta-amyloid 1-40 (2nd year)**	**Δ FDG (delta) vs beta-amyloid 1-40 (baseline)**	**FDG (baseline) vs Plasma tau (baseline)**	**FDG (2nd year) vs Plasma tau (2nd year)**	**Δ FDG (delta) vs Plasma tau (baseline)**
**4 ROIs**	**Male**	p = 0.7177<br>r = -0.0461	p = 0.1557<br>r = 0.1754	p = 0.7863<br>r = -0.0346	p = 0.5954<br>r = 0.0671	p = 0.8899<br>r = 0.0172	p = 0.9443<br>r = -0.0089	p = 0.4977<br>r =-0.0843	p = 0.6697<br>r =-0.0530	***p = 0.0311***<br>***r = -0.2636***
	**Female**	p = 0.5920<br>r = -0.0522	p = 0.3002<br>r = 0.1016	***p = 0.0393***<br>***r = 0.1986***	***#p = 0.0808***<br>***r = -0.1688***	p = 0.5998<br>r = -0.0515	p = 0.7317<br>r = 0.0337	p = 0.5901<br>r = -0.0522	p = 0.2181 r<br>= -0.1189	p = 0.8731<br>r = 0.0155
**Hippocampus**	**Male**	p = 0.5654<br>r = 0.0738	p = 0.9032<br>r = 0.0152	p = 0.3267<br>r = -0.1256	p = 0.5274<br>r = 0.0805	p = 0.9170<br>r = -0.0130	p = 0.5872<br>r = -0.0692	p = 0.6064<br>r = -0.0646	p = 0.1676<br>r = -0.1706	***#p = 0.0728***<br>***r = -0.2223***
	**Female**	p = 0.5777<br>r = -0.0542	p = 0.4924<br>r = 0.0674	p = 0.3267<br>r = -0.1256	***p = 0.0441***<br>***r = -0.1941***	p = 0.8302<br>r = -0.0211	p = 0.3075<br>r = -0.0991	p = 0.3393<br>r = 0.0924	p = 0.7400<br>r = -0.0321	p = 0.7747<br>r = -0.0277
**Para-Hippocampal gyrus**	**Male**	p = 0.5775<br>r = 0.0710	p = 0.7080<br>r = -0.0466	p = 0.5819<br>r = -0.0701	p = 0.7423<br>r = 0.0416	p = 0.9011<br>r = 0.0155	p = 0.9351<br>r = -0.0103	p = 0.5165<br>r = -0.0807	p = 0.3185<br>r = -0.1237	p = 0.1537<br>r = -0.1762
	**Female**	p = 0.9448<br>r = 0.0067	p = 0.4526<br>r = 0.0737	p = 0.1190<br>r = 0.1509	***p = 0.0138***<br>***r = -0.2364***	p = 0.9344<br>r = 0.0081	p = 0.1691<br>r = -0.1333	p = 0.4249<br>r = 0.0772	p = 0.7856<br>r = -0.0264	p = 0.6115<br>r = 0.0492
**Medial frontal cortex**	**Male**	p = 0.5638<br>r = 0.0735	p = 0.1781<br>r = -0.1665	p = 0.6720<br>r = -0.0540	p = 0.1597<br>r = 0.1765	p = 0.6723<br>r = -0.0526	p = 0.8430<br>r = -0.0250	p = 0.5314<br>r = -0.0778	p = 0.6468<br>r = 0.0570	p = 0.6712<br>r = 0.0528
	**Female**	p = 0.2676<br>r = -0.1076	p = 0.7465<br>r = 0.0318	p = 0.1001<br>r = 0.1591	p = 0.1104<br>r = -0.545	p = 0.5011<br>r = -0.0661	p = 0.8089<br>r = -0.0235	p = 0.2785<br>r = -0.1047	p = 0.7138<br>r = -0.0355	***#p = 0.0879***<br>***r = 0.1643***
**Inferior temporal gyrus**	**Male**	p = 0.9524<br>r = 0.0076	p = 0.1632<br>r = 0.1723	p = 0.7253<br>r = -0.0448	p = 0.9357<br>r = -0.0102	p = 0.6978<br>r = 0.0483	p = 0.7339<br>r = 0.0430	p = 0.5642<br>r = -0.0717	p = 0.3317<br>r = -0.1204	*****p = 0.0048***<br>***r = -0.3404***
	**Female**	p = 0.8705<br>r = -0.0159	p = 0.1325<br>r = 0.1470	***#p = 0.0663***<br>***r = 0.1774***	p = 0.1362<br>r = -0.1443	p = 0.8523<br>r = -0.0183	p = 0.8421<br>r = 0.0194	p = 0.5552<br>r = -0.0571	p = 0.3411<br>r = -0.0921	p = 0.9426<br>r = -0.0070

- The statistical p-values were obtained by partial correlation analysis (covariates: age, PiB-PET SUVR values; #p < 0.1, *p < 0.05, and **p < 0.01). Statistically significant p-values were shown in bold & italic.
- Abbreviations: ROI, region of interest; FDG, fluorodeoxyglucose; delta (Δ), difference between the second and first measurement values

Supplementary Table 3. Neurodegeneration markers and plasma beta-amyloid 42, 40, and tau

Brain ROIs	Gender	Plasma beta-amyloid 1-42: Neurodegeneration (baseline) vs beta-amyloid 1-42 (baseline)	Plasma beta-amyloid 1-42: Neurodegeneration (2nd year) vs beta-amyloid 1-42 (2nd year)	Plasma beta-amyloid 1-42: Δ Neuro-degeneration vs beta-amyloid 1-42 (baseline)	Plasma beta-amyloid 1-40: Neurodegeneration (baseline) vs beta-amyloid 1-40 (baseline)	Plasma beta-amyloid 1-40: Neurodegeneration (2nd year) vs beta-amyloid 1-40 (2nd year)	Plasma beta-amyloid 1-40: Δ Neuro-degeneration vs beta-amyloid 1-40 (baseline)	Plasma tau: Neurodegeneration (baseline) vs tau (baseline)	Plasma tau: Neurodegeneration (2nd year) vs tau (2nd year)	Plasma tau: Δ Neuro-degeneration vs tau (baseline)
**Hippocampal volume**	**Male**	p = 0.8626<br>r = 0.0226	p = 0.3469<br>r = -0.1167	p = 0.2300<br>r = -0.1560	p = 0.9398<br>r = 0.0098	p = 0.9278<br>r = -0.0113	p = 0.4950<br>r = -0.0882	p = 0.8928<br>r = 0.0172	p = 0.4746<br>r =-0.0888	p = 0.9061<br>r = -0.0150
	**Female**	p = 0.1728<br>r = -0.1396	p = 0.4394<br>r = -0.0763	p = 0.9426<br>r = 0.0074	***#p = 0.0917***<br>***r = -0.1722***	p = 0.7392<br>r = -0.0329	p = 0.6726<br>r = -0.0434	p = 0.4984<br>r = 0.0691	p = 0.1471<br>r = -0.1404	p = 0.3978<br>r = -0.0864
**Cortical thickness**	**Male**	p = 0.8658<br>r = 0.0219	p = 0.5546<br>r = 0.0735	p = 0.5984<br>r = 0.0682	p = 0.3337<br>r = -0.1238	p = 0.6049<br>r = -0.0488	p = 0.9130<br>r = -0.0141	p = 0.1654<br>r = -0.1741	p = 0.5187<br>r = -0.0802	p = 0.9464<br>r = -0.0085
	**Female**	p = 0.8479<br>r = 0.0192	p = 0.1519<br>r = -0.1408	p = 0.2440<br>r = -0.1164	****p = 0.0141***<br>***r = -0.2425***	p = 0.4953<br>r = -0.0673	p = 0.3701<br>r = -0.0897	p = 0.5611<br>r = -0.0579	****p = 0.0229***<br>***r = -0.2189***	p = 0.6282<br>r = 0.0483

- The statistical p-values were obtained by partial correlation analysis (covariates: age, PiB-PET SUVR values; #p < 0.1, *p < 0.05, and **p < 0.01). Statistically significant p-values were shown in bold & italic.
- Abbreviations: MRI, magnetic resonance imaging; delta (Δ), difference between the second and first measurement values

Supplementary Table 4. Details of ROC curve analyses

	Diagnostic model				Predictive model			
	Male		Female		Male		Female	
**Characteristics (n)**	CV	CV + beta-amyloid 42/40	CV	CV + beta-amyloid 42/40	CV	CV + beta-amyloid 42/40	CV	CV + beta-amyloid 42/40
**AUC (95% CI)**	0.607 (0.48, 0.72)	0.610 (0.48, 0.73)	0.665 (0.57, 0.75)	0.712 (0.62, 0.80)	0.697 (0.57, 0.80)	0.709 (0.58, 0.81)	0.671 (0.58, 0.76)	0.719 (0.63, 0.80)
**Significance (P)**	**0.1325**	**0.1273**	**0.0017 (**)**	**< 0.0001 (****)**	**0.0094 (**)**	**0.0051 (**)**	**0.0102 (*)**	**0.0004 (***)**
**Sensitivity (%)**	94.7	81.6	60.7	75.0	78.6	85.7	75.0	85.0
**Specificity (%)**	30.0	46.7	66.0	62.3	65.4	65.4	58.9	58.9
**+PV / -PV (%)**	63.2 / 81.8	66.0 / 66.7	65.4 / 61.4	67.7 / 70.2	37.9 / 91.9	40.0 / 94.4	28.8 / 91.4	31.5 / 94.6
**Cut-off (Youden index)**	> 0.553	> 0.592	> 0.642	> 0.630	> 0.225	> 0.230	> 0.167	> 0.151

- AUC, area under the curve; 95% CI, 95% confidence interval; cut-off criterion was determined by Youden index

- The cut-off values were logit values derived from logistic regression models with the controlling for ApoE, age, education, and cerebral amyloid deposition (PiB-PET SUVR values)

Supplementary Figures

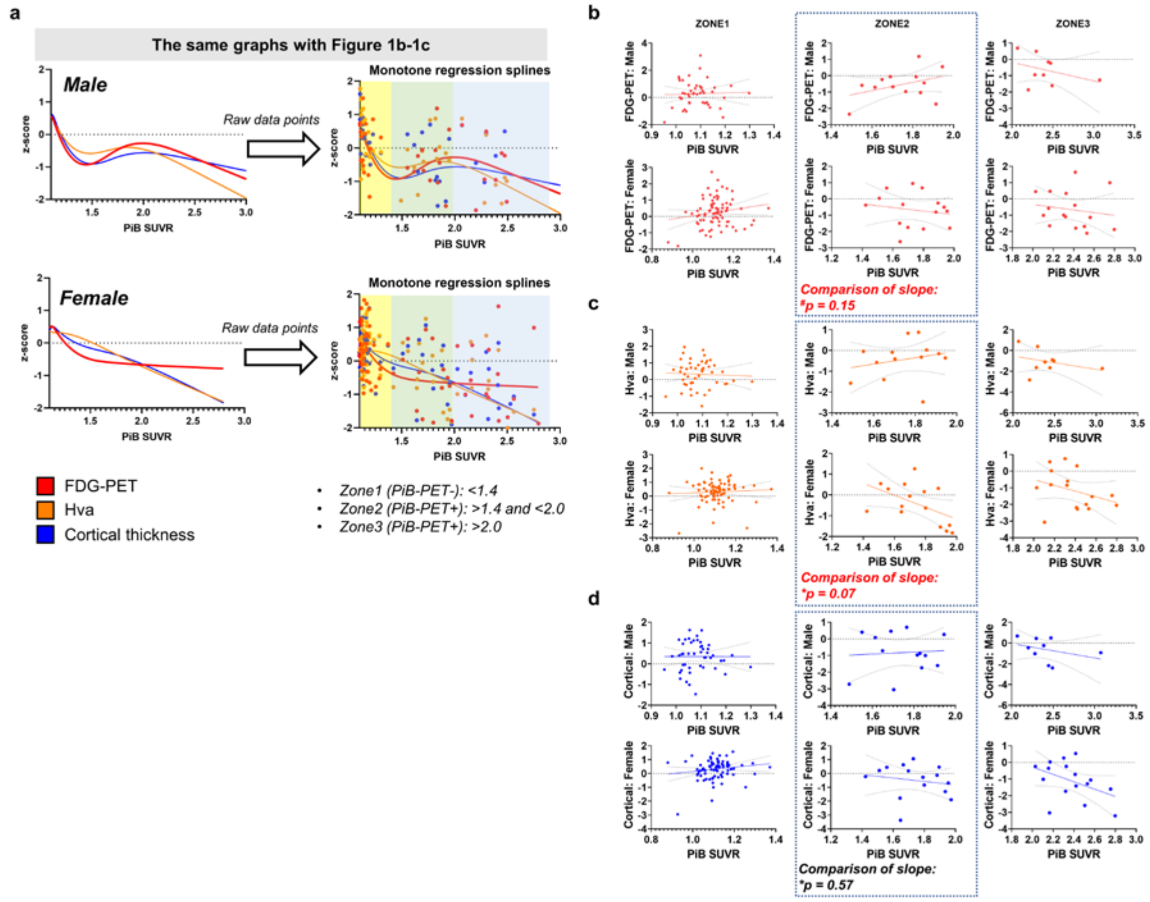

Supplementary Fig. 1. Comparison of zones on the spline curves between males and females (a) The same graph with Figure 1b-1c, but graphs are divided into three zones according to the degree of cerebral amyloid deposition (zone 1, PiB-PET SUVR < 1.4; zone 2, 1.4 ≤ PiB-PET SUVR < 2.0; zone 3, PiB-PET SUVR ≥ 2.0). **(b-d)** Correlation of FDG-PET, hippocampal volume, and cortical thickness with cerebral amyloid deposition depending on each zone. Comparison of slope analysis was performed for zone 2 using Two slopes calculator software (https://www.danielsoper.com/statcalc; $*P < 0.1$, $^{\#}P < 0.2$). **Abbreviations:** PiB-PET, Pittsburgh compound B-positron emission tomography; SUVR, standardized uptake value ratio; Hva, hippocampal volumes; FDG-PET, fluorodeoxyglucose-PET.

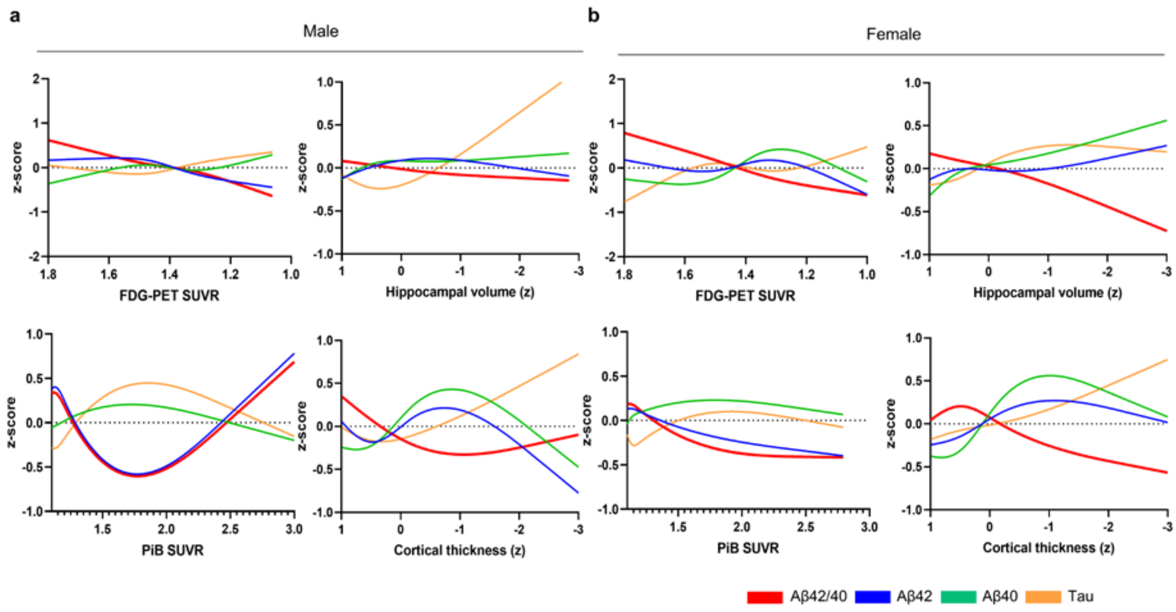

Supplementary Fig. 2. Monotone spline models for plasma biomarkers and brain images (a-b) Monotone spline models for plasma biomarkers in both males and females. To effectively show comparisons between different plasma biomarkers, their levels have been transferred to z-scores. FDG-PET SUVR acted as a proxy for the progression time of brain hypometabolism. PiB-PET SUVR acted as a proxy for the progression time of AD. Hippocampal volume or cortical thickness acted as a proxy for the progression time of neurodegeneration. Smoothing splines with four knots were created. **Abbreviations:** PET, Pittsburgh compound B-positron emission tomography; SUVR, standardized uptake value ratio; Hva, hippocampal volumes; FDG-PET, fluorodeoxyglucose-positron emission tomography.

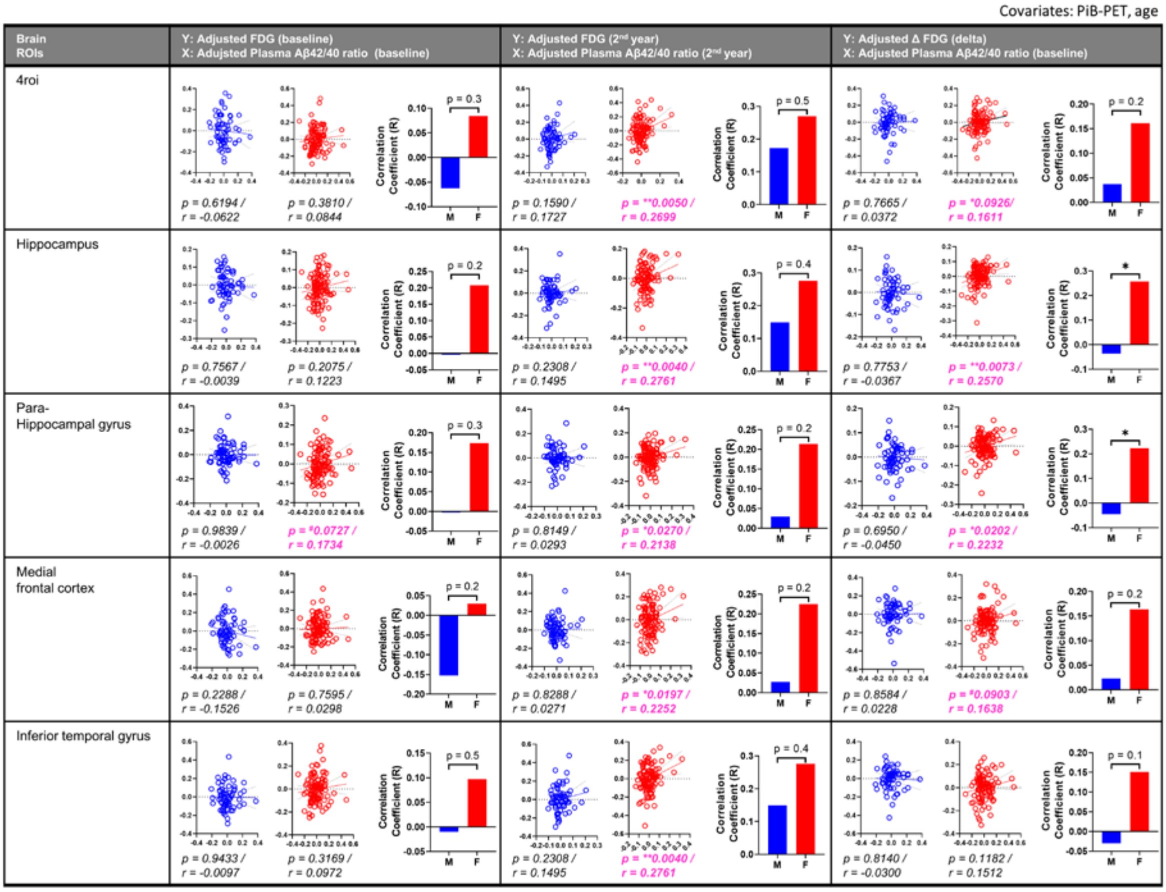

Supplementary Fig. 3. Partial correlation analysis for FDG-PET and plasma beta-amyloid 42/40 ratio and comparison of correlation coefficient (R) between males and females Differences in correlation patterns between sexes. Partial correlation analyses between the plasma beta-amyloid 42/40 ratio and the FDG-PET SUVR values of each region (4rois, composite of angular gyrus, posterior cingulate cortex, precuneus, and inferior temporal gyrus). PiB-PET SUVR values and age were used as covariates. Delta indicates the difference between the first and second measurement values. Comparison of correlation coefficients analyses with the correction of the number of patients between males and females were also performed (*P < 0.1). **Abbreviations:** roi, region of interest; FDG-PET, fluorodeoxyglucose-positron emission tomography; M, males; F, females.

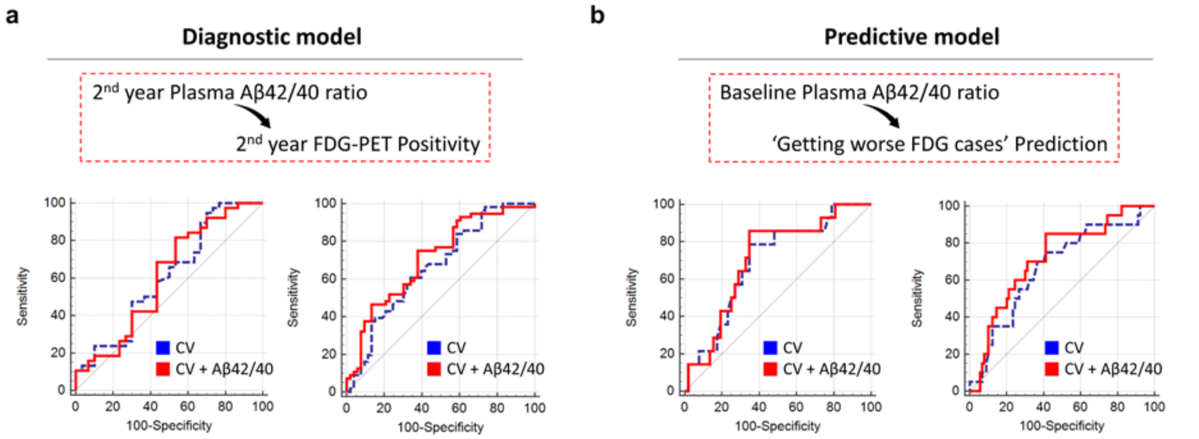

a – Diagnostic model

M	AUC /SE	Sen /Spe	95%CI	P
CV	0.61 /0.07	94.7 /30.0	0.48 ~0.72	n.s. 0.1325
CV + Aβ42/40	0.61 /0.07	81.6 /46.7	0.48 ~0.73	n.s. 0.1273

F	AUC /SE	Sen /Spe	95% CI	P
CV	0.67 /0.05	60.7 /66.0	0.57 ~0.75	** 0.0017
CV + Aβ42/40	0.71 /0.5	75.0 /62.3	0.62 ~0.80	**** <0.0001

Covariates (CV): ApoE, age, education

b – Predictive model

M	AUC /SE	Sen /Spe	95% CI	P
CV	0.70 /0.08	78.6 /65.4	0.57 ~0.80	** 0.0094
CV + Aβ42/40	0.71 /0.07	85.7 /65.4	0.58 ~0.81	** 0.0051

F	AUC /SE	Sen /Spe	95%CI	P
CV	0.67 /0.07	75.0 /58.9	0.58 ~0.76	* 0.0102
CV+ Aβ42/40	0.72 /0.06	85.0 /58.9	0.63 ~0.80	*** 0.0004

Covariates (CV): ApoE, age, education

Supplementary Fig. 4. Plasma beta-amyloid 42/40 ratio and its application in brain metabolic dysfunction biomarker analysis in females (a) Diagnostic ROC curve model for brain metabolic dysfunction. FDG-PET positivity was used as an independent variable based on previous research. The 2nd year plasma beta-amyloid 42/40 ratio values were used as a dependent variable. **(b)** Predictive ROC curve model for brain metabolic dysfunction. The 'getting worse' group, which is the same in Figure 1, was used as an independent variable. Baseline plasma beta-amyloid 42/40 ratio values were used as a dependent variable. ApoE, age, and education levels were used as covariates (CV). *P <0.05, **P <0.01, ***P <0.001, and ****P <0.0001 for the AUC of ROC curves. **Abbreviations:** PiB-PET, Pittsburgh compound B-positron emission tomography; SUVR, standardized uptake value ratio; adj, adjusted; AUC, area under the curve; Sen, sensitivity, Spe, specificity; CI, confidence interval; P, P-values.

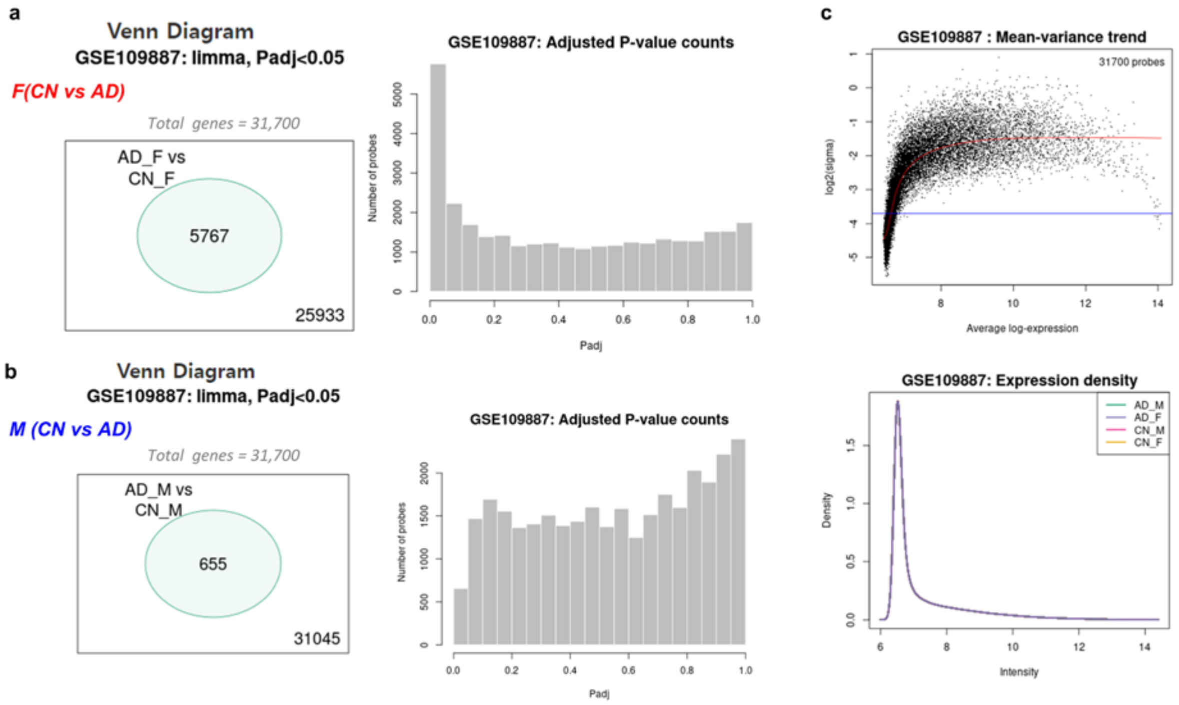

GSE109887

Supplementary Fig. 5. Analysis information for GSE109887 (cohort 2) (a-b) Venn diagram shows that total gene numbers used for RNA sequencing analysis were exactly equal (31,700) for both males and females. Adjusted P-value histograms are also shown for both males and females. **(c)** For RNA sequencing analysis, 31,700 genes were successfully loaded. The expression densities were well distributed for all four groups (no differences between the groups). **Abbreviations:** Padj, adjusted p-value; AD, Alzheimer's disease; CN, cognitively normal; M, males; F, females.

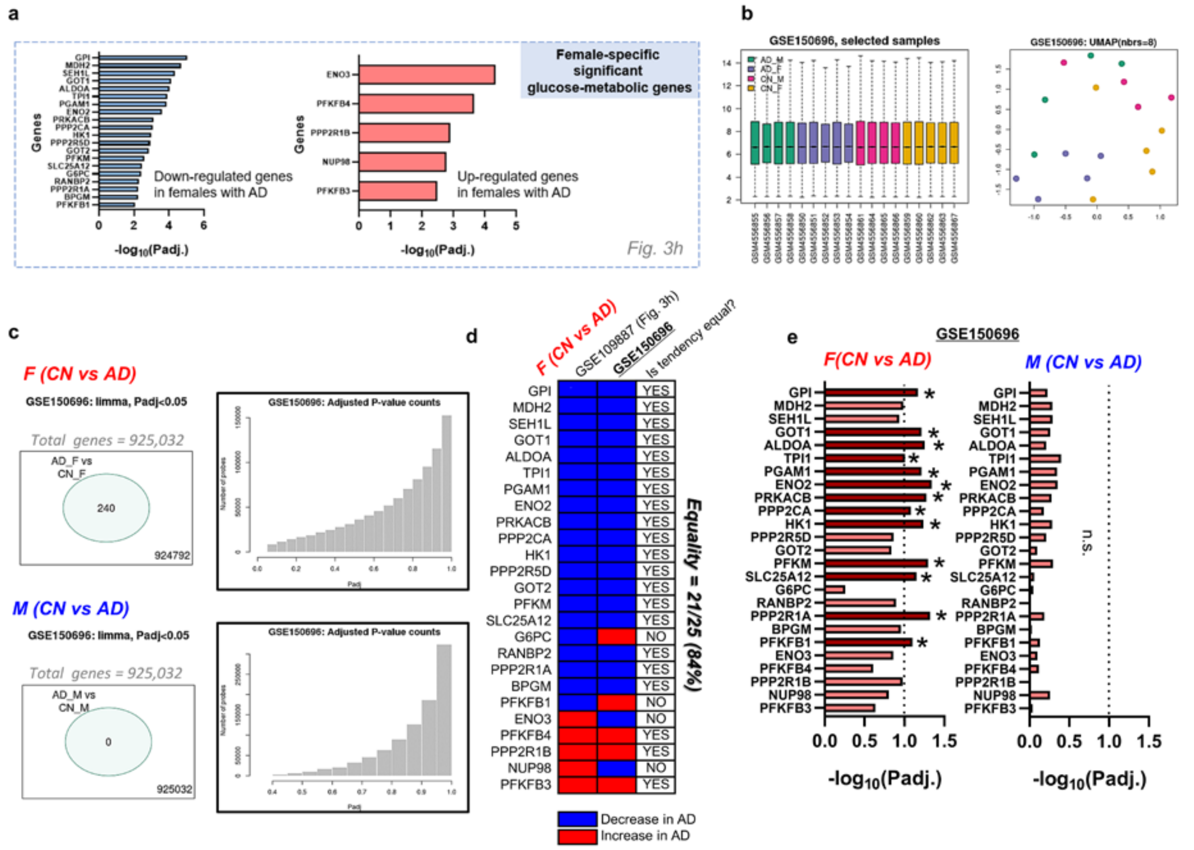

Supplementary Fig. 6. Validation of GSE109887 (cohort 2) using another publicly available cohort (GSE150696; cohort 3) (a) Female-specific glucose metabolic gene list from the result of Fig. 3h. **(b)** Box-plot result shows that the samples were well-normalized and ready for differential expression analysis. Uniform Manifold Approximation and Projection (UMAP) shows how samples are related to each other. **(c)** Venn diagram shows that total gene numbers used for RNA sequencing analysis were exactly equal (925,032) for both males and females. Adjusted P-value histograms are also shown for both males and females. **(d)** Comparison of female-specific glucose metabolic genes between cohort 2 and cohort 3. The up/down tendency for genes from cohort 3 were highly similar to the results from cohort 2 (equality, 84%). **(e)** The female-specific glucose metabolic genes (from cohort 2) also showed statistical significance in females of cohort 3, but not in males. **Abbreviations:** Padj, adjusted p-value; AD, Alzheimer's disease; CN, cognitively normal; M, males; F, females.
